# Differential miRNAs expression pattern of irradiated breast cancer cell lines is correlated with radiation sensitivity

**DOI:** 10.1038/s41598-020-65680-z

**Published:** 2020-06-03

**Authors:** Nastaran Masoudi-Khoram, Parviz Abdolmaleki, Nazanin Hosseinkhan, Alireza Nikoofar, Seyed Javad Mowla, Hamideh Monfared, Gustavo Baldassarre

**Affiliations:** 1 Department of Biophysics, Faculty of Biological Sciences, Tarbiat Modares University, Tehran, Iran; 2 Endocrine Research Center, Institute of Endocrinology and Metabolism, Iran University of Medical Sciences (IUMS), Tehran, Iran; 3 Department of Radiotherapy, Iran University of Medical Sciences (IUMS), Tehran, Iran; 4 Department of Genetics, Faculty of Biological Sciences, Tarbiat Modares University, Tehran, Iran; 5 Division of Experimental Oncology 2, Department of Translational Research, CRO, National Cancer Institute, Aviano, Italy

**Keywords:** Breast cancer, Cancer therapy, miRNAs, Biomarkers

## Abstract

Radiotherapy is a fundamental step in the treatment of breast cancer patients. The treatment efficiency is however reduced by the possible onset of radiation resistance. In order to develop the effective treatment approach, it is important to understand molecular basis of radiosensitivity in breast cancer. The purpose of the present study was to investigate different radiation response of breast cancer cell lines, and find out if this response may be related to change in the microRNAs expression profile. MDA-MB-231 and T47D cells were subjected to different doses of radiation, then MTT and clonogenic assays were performed to assess radiation sensitivity. Cytofluorometric and western blot analysis were performed to gain insight into cell cycle distribution and protein expression. MicroRNA sequencing and bioinformatics prediction methods were used to identify the difference in microRNAs expression between two breast cancer cells and the related genes and pathways. T47D cells were more sensitive to radiation respect to MDA-MB-231 cells as demonstrated by a remarkable G2 cell cycle arrest followed by a greater reduction in cell viability and colony forming ability. Accordingly, T47D cells showed higher increase in the phosphorylation of ATM, TP53 and CDK1 (markers of radiation response) and faster and more pronounced increase in RAD51 and γH2AX expression (markers of DNA damage), when compared to MDA-MB-231 cells. The two cell lines had different microRNAs expression profiles with a confirmed significant differential expression of miR-16-5p, which targets cell cycle related genes and predicts longer overall survival of breast cancer patients, as determined by bioinformatics analysis. These results suggest a possible role for miR-16-5p as radiation sensitizing microRNA and as prognostic/predictive biomarker in breast cancer.

## Introduction

Breast cancer (BC) is one of leading cause of mortality among women worldwide^1^. Radiotherapy (RT) is currently used as the standard treatment for control of breast cancer^2^. Although up to 83% of early- stage diagnosed breast cancer cases can be treated by postoperative radiotherapy, some patients will suffer from spread metastasis and recurrence after radiation therapy^3^. The failure in RT application might be due to radiation resistance, which caused by remaining the cells have greater tolerance to ionizing radiation (IR) or the intrinsic resistance^4^.

Ionizing radiation can cause direct DNA strand break or producing free radicals, leading to more damages in DNA or other cell components. DNA lesions activate the DNA damage response (DDR), which essential for activation of downstream signaling pathways such as DNA repair, cell cycle arrest or apoptosis^5^. Breast cancer cells respond to radiation in different ways, depending on their different biological and molecular characteristic^6,7^. Understanding the molecular mechanism behind the differential response to radiation is important toward a possible personalized use of radiotherapy in patients with different BC subtype. Furthermore, identification of specific molecular target can be used to better classify BC patients^8^.

Variation in radiation sensitivity could be influenced by gene expression and post translational modification^9^. MicroRNAs (miRNAs) are the group of short noncoding RNA that mostly have negative regulatory effect on gene expression^10^. They can regulate almost all cellular processes including DNA repair, cell cycle arrest, apoptosis, and survival^11^. Not surprisingly, miRNAs have been identified as modulator of radiation responses^12^. The relation between deregulated expression of miRNAs and radiation response has been also observed in breast cancer cells^13,14^. For instance, miR-668 could mediate radiation resistance by targeting the NF-κB pathway^15^ or miR-21 is well-known miRNA involved in radioresistance by regulation of G2/M checkpoint^12^; on the other side, miR-200c could enhance radiation sensitivity through inhibiting expression of DNA repair genes and autophagy^16,17^ and miR-620 was upregulated in irradiated breast cancer cells, and its silencing contributed to reduce cell survival following exposure^18^.

Although many clinical studies have identified the specific miRNAs which associated with radiation sensitivity^19–21^, cell-based studies are needed to better understand the role of specific miRNAs in the response to radiation and possibly verify if they can be used as new biomarkers of radioresistance. In the present study, we aimed to investigate if radiotherapy could commonly alter the expression of selected miRNAs in two breast cancer cell lines, namely MDA-MB-231 and T47D, that have different radiosensitivity.

## Results

### Radiation reduced cell viability and colony forming ability of T47D cells

**Figure 1:**
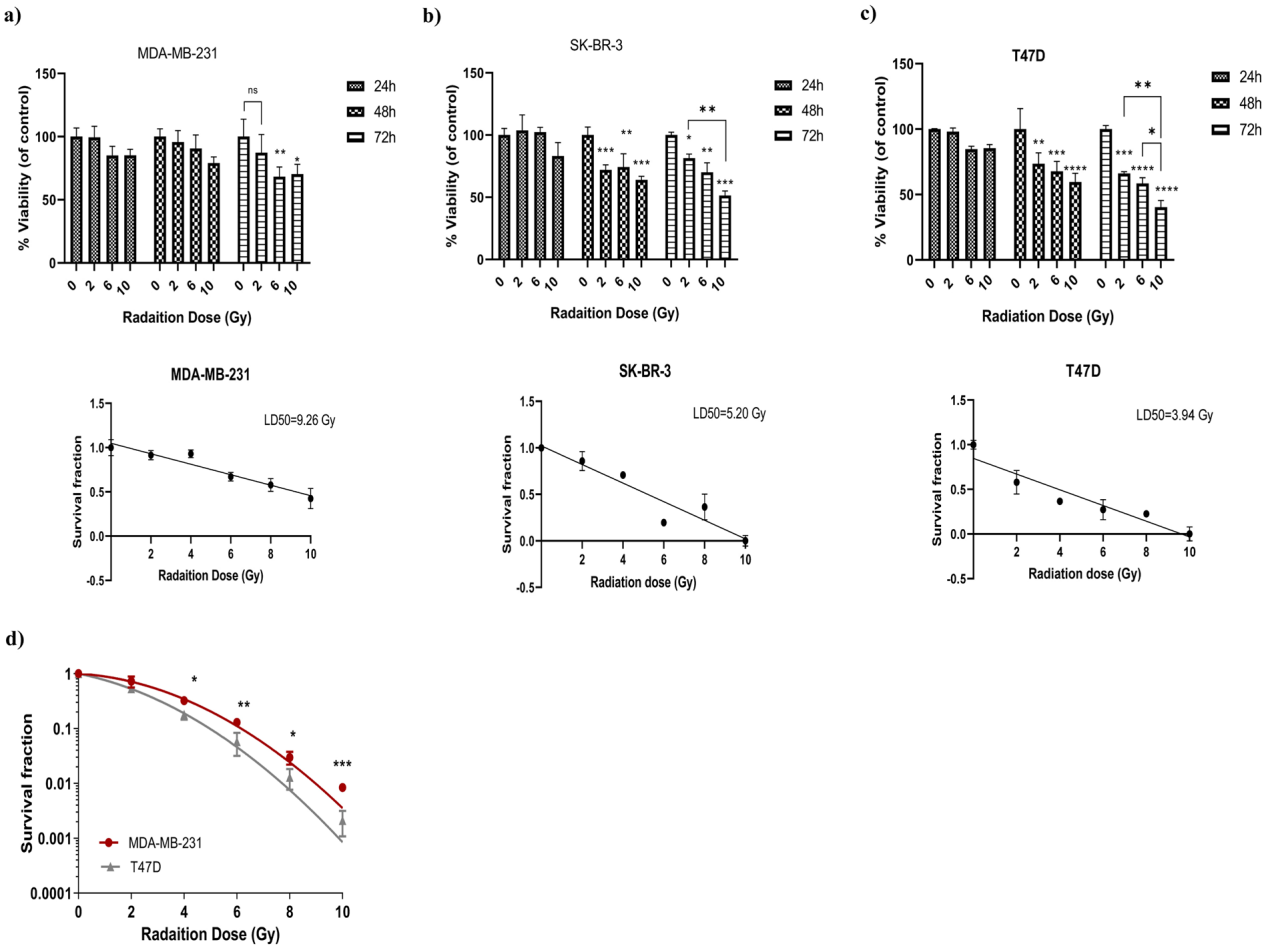
T47D cells are the most sensitive to radiation-induced cell death. (**a–c**) MTT assay evaluating cell viability of MDA-MB-231 (**a**), SKBR3 (**b**) and T47D (**c**) exposed to different radiation doses (2, 6, and 10 Gy) and evaluated 24, 48, or 72 hours after the irradiaition. The LD50 reported in the Fig (a-c). was caculated for each cell line four days after irradiation. Data represent the mean ± standard deviation (SD) of three experiments, each performed in triplcate. Statistical significance was evaluated using two-way analysis of variance (ANOVA), with Tukey’s multiple comparison test. (**d**) Survival curve of MDA-MB-231 and T47D cell evaluated by their colony forming ability using the indicated radiation doses. Data represent the mean ± SD of two independent experiments, each performed in triplicate. Multiple t-test, using the Holm-Sidak method, was used to statistical comparison. *p < 0.05, **p < 0.01, ***p < 0.001 and ns (not significant).

To evaluate radiation sensitivity, three breast cancer cell lines were exposed to increasing dose of radiation and analyzed for cell viability using the MTT assay at different time points after irradiation. As it shown in Fig. 1a, using MDA-MB-231 cells, significant reduction in cells viability was observed only at the doses of 6 and 10 Gy after 72 hours. Conversely, in both SKBR3 and T47D, cell viability gradually decreased with increasing dose of radiation becoming significant after 48 hours of incubation, when compared to non-irradiated control cells (Fig. 1b,c). These differences were more evident in T47D cells as demonstrated by the calculation of their LD_50_ (i.e. the dose of radiation which kills the 50 percent of cells) four days after irradiation. Indeed, the estimated LD_50_ values were of 9.26, 5.20, and 3.94 for MDA-MB-231, SKBR3 and T47D, respectively. Based on these results, we selected the MDA-MB-231 and T47D, as the two cell lines with the highest difference in the response to radiation.

The above data were confirmed by the evaluation of colony forming ability of MDA-MB-231 and T47D irradiated cells showing that the survival fraction of cells exposed to 2 Gy radiation dose was 72.4% and 53.1% for MDA-MB-231 and T47D cells, respectively and that statistically significant differences in cell survival were present in the range of 4 to 10 Gy (Fig. 1d). Cell survival curve was fitted to Linear Quadratic (LQ) model, and α and β parameters were calculated for each cell line. The α represents cell death at low dose, resulting from single radiation event. The β term describes the cell killing at high dose, caused by interaction of damage from two separate radiation events. The higher α and β indicate more radiation sensitivity. The higher α value confirmed the higher radiosentivity of T47D cells, whereas no difference was seen between β values (Table 1).

**Table 1:** Radiobiological parameters of cell survival curve.

	α (Gy^−1^)	β(Gy^−1^)	SF_2_
MDA-MB-231	0.069 ± 0.018	0.049 ± 0.016	0.72 ± 0.069
T47D	0.22 ± 0.0051	0.048 ± 0.0053	0.48 ± 0.089

The α and β describe cell radiosensitivity, cells with higher α or β are more sensitive to radiation.

SF_2_: Survival fraction at dose 2 Gy.

### Induction of G2/M arrest by ionizing radiation in T47D cells

**Figure 2:**
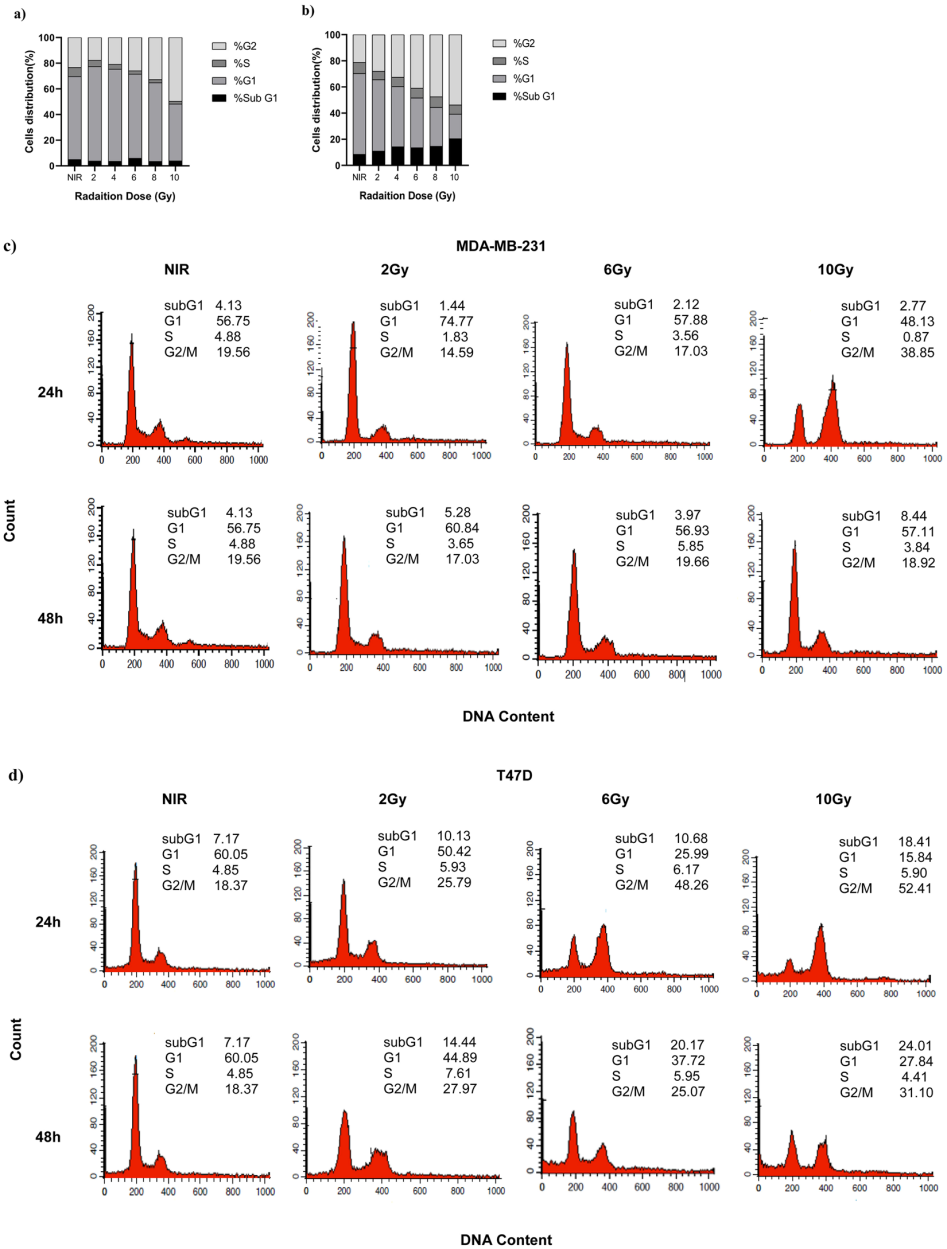
T47D cells display a prolonged G2/M arrest after irradiation. (**a,b**) Graphs reporting the cell cycle distribution of MDA-MB-231 **(a)** and T47D **(b)** treated with 2–10 Gy of radiation and analyzed 24 hours after irradiation. Data represent the mean of two independent experiments. (**c**) Isograms reporting the cell cycle distribution of MDA-MB-231 (**c**) and T47D (**d**) irradiated with 2, 6 and 10 Gy, and analyzed 24 and 48 hours after irradiation. A typical experiment is shown.

We next evaluated the cell cycle distribution of irradiated cells collected 24 and 48 hours after irradiation. As shown in Fig. 2a,b, radiation clearly changed cell cycle distribution in both cell lines, with differences more prominent in T47D compared to MDA-MB-231 cells as evaluated by a dose response curve using of cells collected 24 hours after radiation. In particular, at this time point, T47D cells showed dose-dependent accumulation of the cells in G2/M and subG1 (apoptotic/dead cells) phases of the cell cycle (Fig. 2b). Conversely, at the 24 hours timepoint, MDA-MB-231 cells started to accumulate in the G2/M phase of the cell cycle only at doses ≥6 Gy and failed to accumulate in subG1, again demonstrating a higher resistance to radiation respect to T47D. Next, the analysis of cell cycle distribution over the time demonstrated that independently on the dose used, MDA-MB-231 cells completely recovered from IR within 48 hours with a minimal accumulation of the subG1 population (likely necrotic/apoptotic cells), while T47D cells at the 48 hours timepoint maintained the G2/M block and progressively accumulated in the subG1 phase of the cell cycle (Fig. 2c,d).

### Different expression pattern of cell cycle and DNA repair related proteins in T47D cells

**Figure 3:**
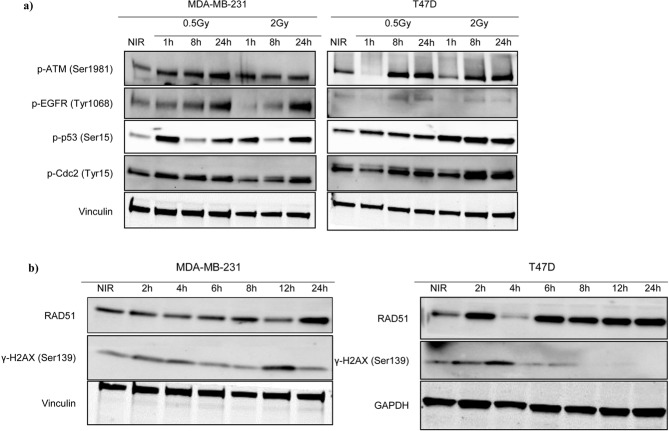
Signal transduction pathways are differently activated by radiation in T47D and MDA-MB-231 cells. (**a**) Western blot analysis evaluating the expression of the indicated phospho-proteins in T47D and MDA-MB-231 irradiated with 0.5 and 2 Gy doses and the harvested at the indicated time points. Vinculin was used as loading control. (**b**) Western blot analysis evaluating the expression of γH2AX and RAD51 in cells irradiated with 2 Gy and then collected at the indicated time points. Full-length blot is shown in Supplementary Fig. S1. Vinculin and GAPDH were used as loading control. NIR = Non-Irradiated cells.

The differences in cell cycle distribution were accompanied by biochemical differences in key signal transduction pathways, as evaluated by western blot looking at the expression of selected phosphorylated proteins. To use a clinical relevant conditions and to better highlight the differences between RT-sensitive and –resistant cells, we used the 2 Gy (dose used in fractionated RT) and the 0.5 Gy doses. Irradiated cells were harvested at different time points (i.e. 1, 8 and 24 hours after radiation) and cell lysates analyzed by western blot. In MDA-MB-231 cells the expression of both pSer1981-ATM and pTyr1068- EGFR, two known mechanisms of resistance to radiation, rapidly increased (onehour timepoint) (Fig. 3a, left panels). The increased expression of pSer1981-ATM remained higher than the one observed in non-irradiated cells up to 24 hours after irradiation, while EGFR phosphorylation even increased over the same period of time (Fig. 3a, left panels). In T47D, ATM phosphorylation increased 8 hours after irradiation both at 0.5 and 2 Gy doses while EGFR phosphorylation was only slightly modified by irradiation at all the considered time points (Fig. 3a Right panels).

The expression of pSer15-TP53 and pTyr15-CDK1, used as markers of cell cycle arrest and apoptosis and G2/M transition respectively, was evaluated on the same lysate. Results show that in MDA-MB-231 cells, irradiation induced a biphasic increase of pSer15-TP53 at one and 24 hours timepoints. These changes were independent on the dose used (Fig. 3a, left panels). The expression of pTyr15-CDK1 (a phosphorylation event that prevents the fully activation of CDK1 and thus, when present, it blocks the cell cycle at the G2/M transition), was only slightly modified by 0.5 Gy dose and started to increase with the 2 Gy dose at 24 hours after treatment (Fig. 3a, left panels). In T47D cells, both the increase in pSer15-TP53 and pTyr15-CDK1 was dose dependent. However, while pSer15-TP53 expression increased after one hour of treatment and remained high over the whole period of observation, with only a small decrease 24 hours after irradiation, pTyr15-CDK1 increased at the 8 hours timepoint and remained high up to 24 hours after treatment (Fig. 3a, right panels), in line with the G2/M accumulation observed in FACS analyses (Fig. 2).

Finally, we evaluated the expression of two markers of DNA damage, namely RAD51 and γH2AX (i.e. Ser139 phosphorylated Histone H2X). In T47D, RAD51 expression increased rapidly and remained high up to 24 hours while γH2AX expression reached a peak 4 hours after irradiation and then rapidly decreased (Fig. 3b, left panels). In MDA-MB-231 cells, the levels of RAD51 increased only at 24 hours post irradiation while γH2AX expression was only slightly modified over the considered period of time (Fig. 3b, right panels).

### MicroRNA expression profiling identified upregulation of miR-23b-3p, miR-16-5p in radiosensitive breast cancer cells

**Figure 4:**
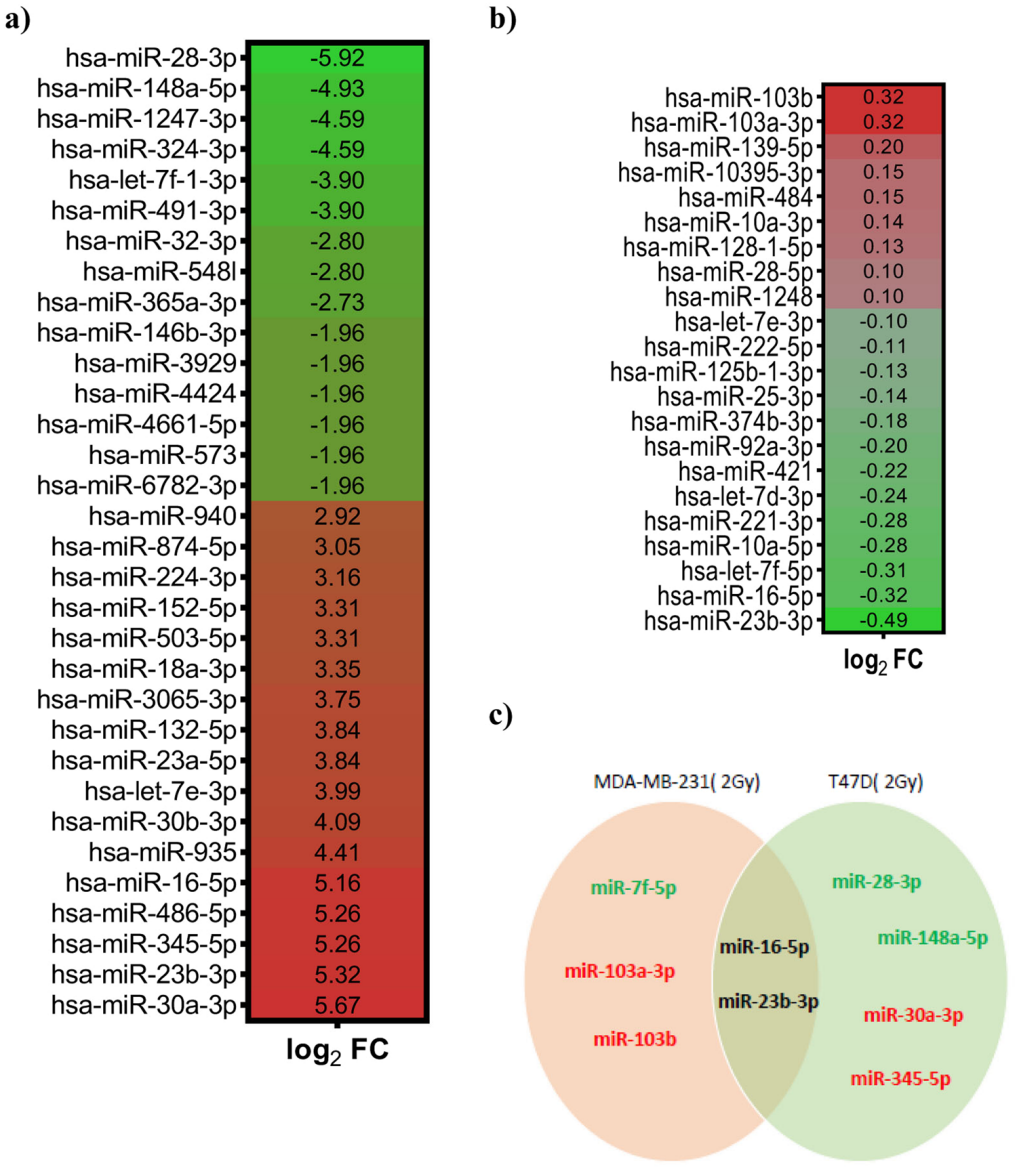
Evaluation of miRNAs expression modified by irradiation in breast cancer cells. (**a,b)** Heat maps of the most differentially expressed miRNAs in T47D **(a)** and MDA-MB-231 **(b)** cells irradiated with 2 Gy dose and analyzed 24 hours after irradiation and compared to the miRNAs expression levels of the respective non-irradiated cells. (**c**) Venn diagram of the 5/6 most differentially expressed miRNAs in the two cell lines. In red are reported miRNAs overexpressed and in green those downregulated in irradiated cells. FC = Fold change.

Data collected so far indicated that T47D and MDA-MB-231 had different sensitivity to irradiation with differences in cell cycle distribution and signaling pathways activation. We thus investigated if these differences were also accompanied by differences in miRNAs expression using microRNAs sequencing. The miRNAs expression level in MDA-MB-231 and T47D irradiated cells collected 24 hours after the treatment was compared with their non-expression in not-exposed control. In MDA-MB-231, we found 88 differentially expressed miRNAs of which 44 were upregulated and 44 were downregulated in irradiated cells. A larger proportion of miRNAs was modified in T47D cells where we found 183 were upregulated and 59 were downregulated by 2 Gy irradiation (Table 2). The most upregulated and downregulated miRNAs in radiation treated T47D and MDA-MB-231 cells are reported in Fig. 4a,b. When we look closer to the expression of the miRNAs mostly modified in the two cell lines, we observed that the miR-23b-3p and miR-16-5p were commonly modified by irradiation being upregulated in T47D and downregulated in MDA-MB-231 (Fig. 4c).

**Table 2:** The deregulated miRNAs in the cell exposed to 2 Gy dose at 24 hours.

DE group	Total DEG	Upregulated	Downregulated
MDA-MB-231 (2 Gy) vs. MDA-MB-231 (Control)	88	44	44
T47D (2 Gy) vs. T47D (Control)	242	183	59

DEG: Differential Expression Group.

### The miR-16-5p showed different expression in radioresistant compared to radiosensitive cells

**Figure 5:**
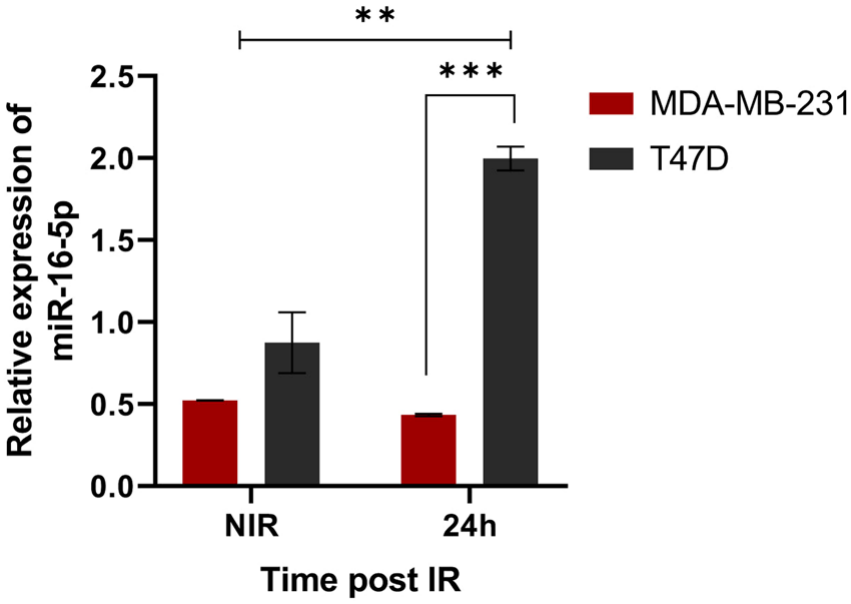
Validation of miR-16-5p expression modification. Graph reporting the normalized expression of miR-16-5p in T47D and MDA-MD-231 cells exposed to 2 Gy and collected after 24 hours evaluated by the qRT-PCR analysis. The U6 (RNU6-1) snRNA was used as reference gene. Data represent the mean ± standard deviation (SD) of two experiments, performed in duplicate. Statistical significance was evaluated using two-way analysis of variance (ANOVA), with Tukey’s multiple comparison test. **p < 0.01, ***p < 0.001.

To validate the miRNA-Seq results and evaluation of the difference in miR-16-5p expression after irradiation, we used quantitative real-time polymerase chain reaction (qRT-PCR) on RNA extracted from both cell lines 24 hours after irradiation with 2 Gy. In line with the miRNA sequencing results, we found a two-fold increase in miR-16-5p expression in irradiated T47D respect to non-irradiated cells, while no significant differences were observed between irradiated and non-irradiated MDA-MB-231 cells (Fig. 5).

### The miR-16-5p targets prediction and pathway enrichment analysis

**Figure 6:**
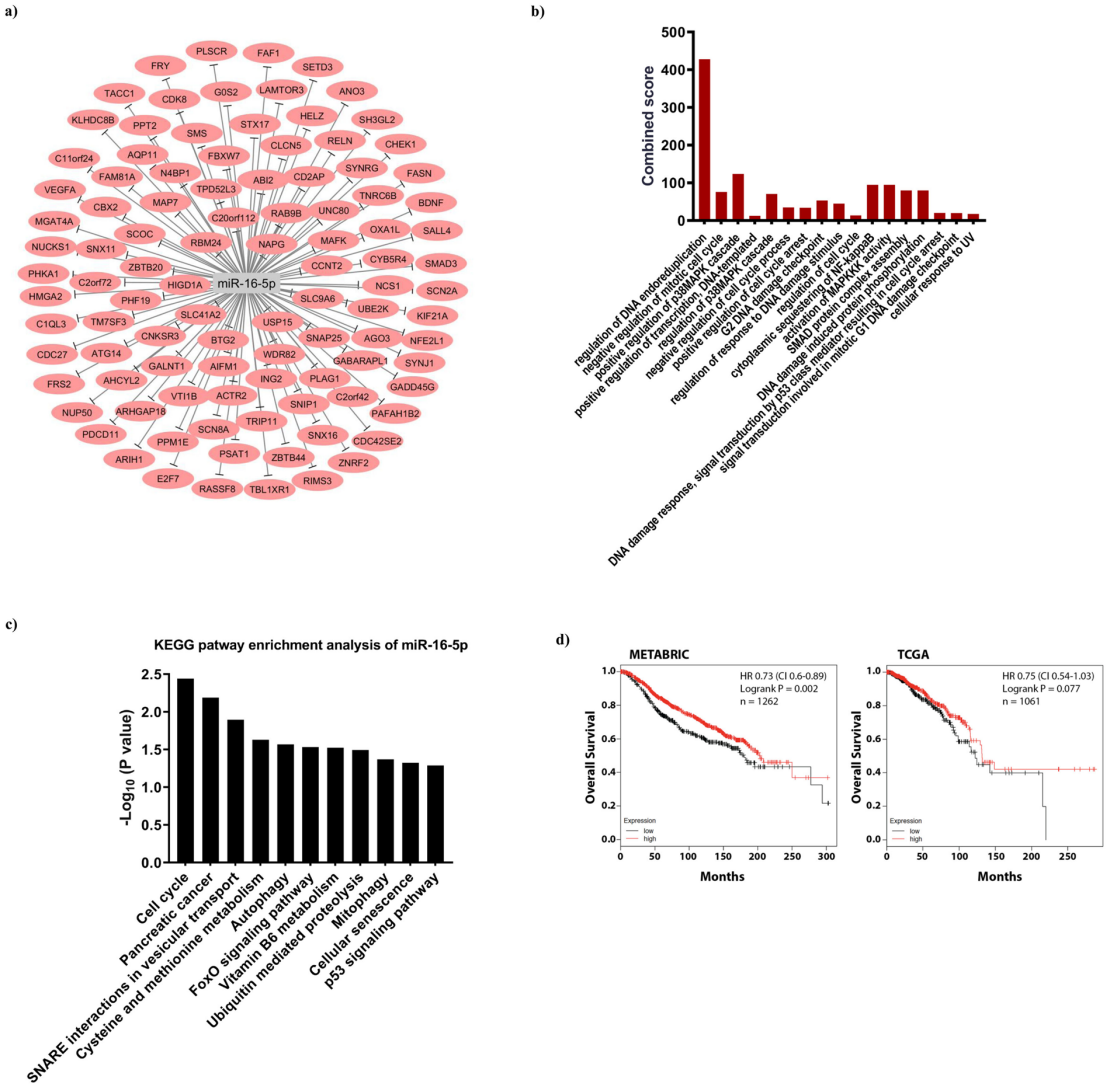
Bioinformatics evaluation of miR-16-5p target genes. (**a**) The predicted genes target of miR-16-5p obtained setting the cut of ≤–0.4 using the target prediction TargetScan V7.2. (**b)** GO biological process and (**c)** KEGG pathway enrichment analysis evaluating the process/pathways most significantly represented among the possible miR-16-5p target genes (as evaluated in a). (**d**) Kaplan-Meier survival curves evaluating the Overall Survival (OS) of stage breast cancer patient included in the METABRIC (n = 1262, left) and TCGA (n = 1061, right) datasets, based on the expression of miR-16 using the KM Plotter online tool. HR = Hazard Ratio and CI = Confidence Interval. p-values were calculated using the log-rank test.

The potential target genes of miR-16-5p were retrieved by online TargetScan database, after setting threshold ranging from −1 to −0.4 for total score and then visual mapped in Cytoscape (Fig. 6a). The repression of the target gens are defined by the complementary features between miRNA and mRNAs, as negative score represent better repression^22^. To explore the probable biological function and related pathway, Gene Ontology and KEGG enrichment analysis were predicted through Enrichr online database. The results showed the correlation between target genes and negative regulation of mitotic cell cycle (GO: 0045930), G2 DNA damage checkpoint (GO: 0031572), regulation of response to DNA damage stimulus (GO: 2001020), activation of MAPKKK activity (GO: 0000185), cellular response to UV (GO: 0034644), DNA damage response, signal transduction by p53 class mediator resulting in cell cycle arrest (GO: 0006977), cytoplasmic sequestering of NF-kappaB (GO: 0007253) and DNA damage induced protein phosphorylation (GO: 0006975). KEGGG pathway analysis also indicated that cell cycle is the downstream pathway principally related to miR-16-5p (Fig. 6b,c).

Finally, using in silico analyses we tried to verify if miR-16 expression, being correlated with a higher sensitivity to radiation, could have a prognostic value in breast cancer patients. To this aim we used the Kaplan- Meier plotter online tool, which allows verifying the prognostic potential of miRNAs in up to four different databases. Our analyses showed that both in the Metabric dataset (n = 1262 patients with a median follow up of 94 months) and in the TCGA dataset (n = 1061 patients with a median follow up of 25 months) high miR-16 expression predicts longer overall survival for the patients (Hazard Ratio 0.73 and 0.75 for Metabric and TCGA, respectively) (Fig. 6d).

## Discussion

Radiation resistance represents a relevant clinical unmet need in the treatment of breast cancer patients. Identification of molecular markers involved in radiation response could help to identify new drugs as a radiosensitizer and could be used as predictive biomarker of radiation response^23^. Several studies have reported many potential targets for enhancement of radiation sensitivity in breast cancer cells^24–27^.

Here we report the identification of miR-16 as a possible prognostic biomarker in breast cancer starting from the analysis of miRNAs modified by radiation in breast cancer cells.

To reach this goal we have characterized an *in vitro* model of radiation response using two estrogen receptors positive and one triple negative breast cancer cell lines. Among the three tested breast cancer cell lines, we selected MDA-MB-231 and T47D cells that showed the highest differences in radiation sensitivity. Using clonogenic assay to extrapolate radiobiological parameters, we found that T47D had a 3.1 folds higher α value accompanied by a 1.5 folds higher SF2 when compared to MDA-MB-231 suggesting that they had an intrinsic radiation sensitivity^28^. Similar results were recently reported by Speers *et al*. that showed a higher survival fraction for MDA-MB-231 compared to T47D cells at 2 Gy dose^29^.

Induction of cell cycle arrest in both G1 and G2 cell cycle phases provide time for DNA damages repair following irradiation^23^. Interestingly, we found a stronger increase of G2/M cell population in T47D compared to MDA-MB-231 cells in each dose of radiation. This result is in agreement with the previous findings reporting that radiation-induced G2 arrest is more pronounced in radiosensitive respect to radioresistant cells^30^. These differences are in line with the notion that in response to radiation cancer cells usually activate G2 checkpoint to complete DNA repair.

Following irradiation G2 cell cycle arrest is regulated by activation of ATM-CHK2 pathway that eventually induce the phosphorylation of cyclin- dependent kinase like CDK1 (CDC2) on Tyr-15 by WEE1 kinase, preventing CDK1 full activation and inhibiting G2/M transition^31^. Accordingly, we found in T47D a higher radiation-dependent CDK1 phosphorylation that might explain the higher percentage of G2 arrested cells in T47D respect to MDA-MB-231.

The tumor suppressor gene TP53 is a validated target of ATM that phosphorylates p53 protein on Ser15^32^. This is an activating phosphorylation that increases p53 transcriptional activity that eventually participates in the establishment of the G2 checkpoint following irradiation^33^. Accordingly, we found that in both T47D and MDA-MB-231, p53-Ser15 is phosphorylated although with different kinetics, which might reflect the different G2/M arrest observed in the two cell lines. Of note, both T47D and MDA-MB-231 carried a mutated TP53 that however could also sustain the radiation-induced G2 arrest^34^.

EGFR expression and phosphorylation has been associated with decreased efficacy of radiotherapy not only in Head and Neck Squamous Carcinoma but also in TNBC cells^35,36^. In our study, the high expression of phosphosho-EGFR was observed in MDA-MB-231, but not in T47D cells supporting the possibility that the higher radiation resistance of MDA-MB-231 could be at least partially due to EGFR phosphorylation.

The different activation of signal transduction pathways was also followed by a different expression of γH2AX and RAD51, whose persistent expression has been linked to un-rejoined DSB and increased radiosensitivity^37^.

Interestingly, the different biological and biochemical response of MDA-MB-231 and T47D cells allowed us to identify miR-16 as a possible important mediator of response to radiation.

Of course it is possible that other differentially expressed miRNA (e.g. miR-23b-3p) could participate to the response to radiation. In the available literature the role of miR-23b-3p in the response to irradiation is still controversial and not investigated in breast cancer^38,39^, therefore it could be relevant to explore its role in breast cancer response to RT in future studies.

The miR-16, which belongs to miR-15/miR-16 cluster, is the example of highly conserved miRNAs able to regulate several important signaling pathway like cell proliferation, apoptosis and cell cycle^40^. In the context of breast cancer miR-16 has been reported to be down regulated respect to normal breast tissues with the lowest expression observed in highly metastatic breast cancer^41,42^.

Yet, only few other reports have investigated the role of miR-16 in the response to radiation, showing that it could promote the sensitivity to radiation of non-small cell lung and breast cancer^43,44^. Moreover, miR-16 was also identified as the regulator of immune response tumor microenvironment after radiation therapy^45^. In this context it is plausible that miR-16 might mediate radiation-induced G2 arrest by the targeting of cell cycle regulators like WEE1 and/or CHEK1 that control the G2/M transition and are validated miR-16 target genes. Intriguingly, by bioinformatics we also observed that high miR-16 predicts good prognosis in breast cancer patients.

This study has some limitations. First, although our bioinformatics analyses indicated that miR-16-5p may also be involved directly in the regulation of genes, those involved in radiation sensitivity include CHEK1, CDC27, SMAD3, GADD45G we did not prove in wet lab experiments that these genes are bona fide miR-16 targets in control and/or irradiated cells. Similarly, we did not prove that miR-16-5p act as radiosensitizer by modifying its expression in radioresistant and in radiosensitive cells and therefore it is possible that the increased expression of miR-16 in T47D cells after irradiation is only an epiphenomenon and it does not have a causal role of the increased radiosensitivity of these cells. We hope we will be able to better investigate and clarify these points in future studies.

Despite these limitations, our work starting from the analyses of the biological response of different breast cancer cell lines to radiation leaded to the identification of miR-16-5p as radiation modified miRNA and as biomarker of breast cancer patient’s prognosis, and possible radiosensitivity. Future works on larger cohorts of breast cancer patients are necessary to confirm this possibility.

## Methods

### Cell culture and radiation exposure

The breast cancer cell lines MDA-MB-231, T47D, and SKBR3 were grown in Dulbecco’s Modified Eagle’s Medium (DMEM) (Gibco) and RPMI1640 (Roswell Park Memorial Institute medium) (Gibco) containing 100 units/mL of penicillin and 100 mg/mL of streptomycin, supplemented with 10% Fetal Bovine Serum (FBS) (Gibco), and maintained in humidified 37 °C incubator with 5% CO2. Cells were seeded at different density, and incubated for 24 hours to reach 60–70% confluence for all experiments. Then, cells were exposed to different doses of radiation 2, 4, 6, 8, and 10 Gy, using Elekta Compact Linear Accelerator, with 6 MeV and dose rate 2 Gy/ min. The medium was changed immediately after irradiation.

### Cell viability assay

The MTT (3-(4,5-Dimethylthiazol-2-yl)-2,5-diphenyltetrazolium bromide) cell cytotoxicity assay was performed as previously described^46^. Briefly, cells (8 × 10^3^ cells/well) were cultured in 96-well plate, 24 hours before treatment. Cells were irradiated by different doses of radiation (2 to 10 Gy). After incubation for 24, 48, 72 and 96 hours, 10 µl MTT (5 μg/ml) was added to each well followed by incubation for 3 hours. Next 100 µl DMSO was added to dissolve precipitation, and kept at room temperature for 10 minutes with gentle shaking. Non-irradiated cells were treated as same procedure. The absorbance was quantified by microplate reader (BioTek Instruments, Inc., Winooski, VT, USA) at 540 nm. The viability was normalized to non- irradiated group.

### Clonogenic assay

The breast cancer cells were seeded in 6-well plates and let to adhere to the plates. Cells were exposed to different doses of radiation (2 to 10 Gy). Then cells were plated in triplicate in 6-well plate and maintained at 37 °C and 5% CO_2_ for 7–14 days. The cells were fixed with 4% (v/v) paraformaldeide, and stained with 5% (w/v) crystal violet. Colonies with more than approximately 50 cells were counted manually, and clonogenic survival fraction (SF) was expressed as the relative plating efficiencies of the irradiated cells to the control cells. The linear-quadratic model (LQ model) was used to describe cell survival fraction as the function of radiation dose. The survival fraction was fitted into inverse exponential equation:

$${\rm{SF}}=\exp (-{\rm{\alpha }}{\rm{D}}-{{\rm{\beta }}{\rm{D}}}^{2})$$

In this equation, SF is surviving fraction of cells at dose D, and α and β are two constants of linear and quadratic components of cell killing^28^.

### Cell cycle analysis

Cell cycle distribution was examined by Propidium Iodide (PI) staining according to previous protocol^47^. Briefly, 2 × 10^5^ cells were seeded per well in 6-wells plates, and allowed to attach for 24 hours. Plates were then exposed to different doses of radiation and the medium was replaced immediately after irradiation. Irradiated cells were collected 4 and 24 hours after irradiation by tryspinization. Cell suspension was washed with ice-cold PBS twice, and fixed by adding 70% (v/v) cold ethanol and under gentle agitation. The cells were then maintained in 4 °C in the dark until used. Prior to staining, fixed cell were washed twice in PBS, resuspended in PBS supplemented with 100 μg/mL RNase A, 50 μg/mL propidium iodide and 0.1% (v/v) Triton X-100 and then incubated at 37 °C for 30 minutes, protected from light. DNA content was quantified by measuring PI florescence signal versus count, using FACSCalibur Flow Cytometer (BD, San Jose, CA, USA). The percentage of cell in each phase of the cell cycle was analyzed using the FACSDiva 7.0 software (BD Biosciences).

### RNA extraction and sequencing

For RNA extraction, cells in exponential growth phase were subjected to irradiation with a 2 Gy dose, replacing medium and incubated for additional 24 hours. The total RNA was extracted using TRIzol total RNA isolation reagent (Invitrogen)and Total RNA isolation kit (Norgen Biotek Corporation, Canada) according to manufacturer’s instruction. The concentration of RNA was more than 500 ng/µl for all samples with an OD260/OD280 ratio equal to 2 and more, as quantified by NanoDrop spectrophotometer (NanoDrop Technologies, Wilmington, DE, USA). The RNA integrity was also checked by agarose gel electrophoresis. For small RNA sequencing, total RNA was subjected to ligation 3′ and 5′ small RNA adapters. Then, single strand cDNA was synthesized and amplified using specific primers followed by purification and quantification. The small RNA libraries were loaded on flow cell and then sequenced on Illumina HiSeq2000 genome analyzer, using SBS (sequencing by synthesis) method according to manufacturer’s instructions^48–50^.

### miRNA-Seq analysis pipeline

FASTQ files were groomed and subsequently FASTQC algorithm was applied to check the presence and the type of adaptor contaminations within FASTQ files. Trimming of FASTQ files were then carried out using Trim Galore algorithm^51^ followed by running FASTQC^52^ to ensure removing of adaptor contaminations. The transcriptome reference sequence was downloaded from miRBase database, (Version 22). Mapping of short reads on the reference transcriptome reference sequence were performed applying HISAT2 algorithm^53^. Read counts for each feature were calculated using Salmon algorithm^54^. Finally, DESeq2 were employed on the resulted read counts to get the list of deregulated miRNAs. Applying the adjusted p-value ≤0.05 the most significant up and down-regulated genes were selected for downstream studies.

### Western blot

To evaluate protein expression, western blot analysis was performed on protein extract of cells, were exposed to 0.5 and 2 Gy radiation doses at different time points. For cell lysate preparation, cells were washed with cold PBS twice, collected in 100 µl cold RIPA lysis buffer (50 mM Tris HCl pH 8, 1% Igepal, 0.5% sodium deoxycholate, 0.1% SDS), containing protease inhibitor cocktail (Complete TM, Roche), 1 mM Na3VO4 (Sigma), 100 mM NaF (Sigma) and 1 mM DTT (Sigma). Total protein concentration was quantified by standard Bradford assay (Bio-Rad; CA, USA). 25 to 40 μg of proteins were mixed with 5 µl of Laemmli sample buffer and boiled at 95 °C for 5 minutes. The protein samples were then loaded and separated using 4–20% SDS polyacrylamide precast gels (Criterion Precast Gel, Bio-Rad). Proteins were transferred to nitrocellulose membranes (GE Healthcare), and membrane blocked with 5% Non-Fat Dried Milk (NFDM) in Tris Buffered Saline-Tween (TBS) containing 0·1% Tween-20 (TBST) for 1 hour at room temperature (RT). Blocked membranes were incubated overnight at 4 °C using the following primary antibodies: phospho-ATM (Ser1981) (1:1000, #13050), phospho-EGFR (Tyr1068) (1:1000, #3777), phospho- p53(Ser15) (1:500, #9284), phospho- cdc2(Tyr 15)(1:1000, #9111), Rad51 (D4B10)(1:1000 #8875) were from Cell Signaling Technology, γH2AX(Ser139)(1:1000) and GAPDH (6C5 CB1001, 1:1000) were from Millipore, and goat polyclonal antibody against vinculin (1:5000, sc-7649 clone N-19) was from Santa Cruz Biotechnology.

After incubation with the primary antibodies membranes were washed in TBST (x3), and incubated with secondary antibodies at RT for 1 hour. The Horseradish peroxidase-conjugated goat anti-mouse and goat anti-rabbit antibodies (Rockland) were prepared in 5% NFDM in TBST at the concentration of 1:2000. The fluorophores-conjugated antibody (AlexaFluor 680, Invitrogen or IRDye 800, Rockland) was diluted 1:1500 in Odyssey Blocking Buffer (LI-COR, Biosciences). Finally, proteins expression were detected by Enhanced Chemi -Luminescence (ECL) system or Odyssey Imaging scanner (LI-COR), as appropriate. To evaluate the quantity and quality of loaded proteins, we used Vinculin and GAPDH as loading controls. The membranes were stripped using the Re-blot Plus Strong Solution 10×(Millipore) diluted 1:10 in ionized water for 10 minutes at room temperature. After washing three times in TBST, the membranes were reblot as described above.

### Quantitative real-time PCR (qRT-PCR)

Total RNA from 2 Gy irradiated breast cancer cells was extracted using TRIzol (Invitrogen) 24 hours after irradiation, then quantified and diluted to reach the concentration of 50 nanogram per each µliter. The concentration of RNA was measured by NanoDrop 3300 spectrometer (Thermo Scientific, Waltham, MA, US). The cDNA was produced by TaqMan MicroRNA Reverse Transcription Kit (Applied Biosystem) as previously described^55^. Following cDNA dilution, qRT–PCR was carried out using TaqMan microRNA assay kit (Applied Biosystems) using the CFX96 Thermocycler (Bio-Rad, USA). U6 (RNU6-1) snRNA was used to normalize miRNAs expression.

### miRNAs target prediction and pathway enrichment analysis

The target prediction of differentially expressed miRNA was performed using TargetScan 7.2 bioinformatics software tool (http://www.targetscan.org/vert_72/)^56^. The predicted genes were selected by setting the threshold based on the total context score ≤ −0.4. Then the genes information were uploaded to Enrichr online database (https://amp.pharm.mssm.edu/Enrichr/) to predict target related pathways throughout Gene Ontology (GO) analysis tool and Kyoto Encyclopedia of Genes and Genomes (KEGG) pathway^57,58^. We specifically focused on targets involved in DNA repair, cell cycle and apoptosis regulation.

### Statistical analysis

Statistical analyses were performed using GraphPad PRISM, Version 8 (GraphPad Software Inc. CA) and the difference with p-value below 0.05 was considered significant. The radiobiological parameters of cell survival curve were also measured by GraphPad prism.

The LD50 values were estimated using the equation of first order polynomial non-linear regression. Western blot images were analyzed using ImageLab analysis software, Version 6 (Bio-Rad, Hercules, CA). Kaplan-Meier survival curves were generated using the KM Plotter online tool (http://kmplot.com)^59^, using the most appropriate cut off choice. KM Plotter is an online algorithm exploitable to interrogate the expression of up to 1052 miRNAs on up to 1,262 (METABRIC dataset) or 1061 breast cancer patients (TCGA dataset) with a mean follow-up of 94 (METABRIC dataset) or 25 months (TCGA dataset), as described previously^60,61^.

## Supplementary information

Supplementary information.
