# Supplementary material for: Differential miRNAs expression pattern of irradiated breast cancer cell lines is correlated with radiation sensitivity
Source: Sci Rep. 2020 Jun 3;10:9054. doi: 10.1038/s41598-020-65680-z (PMC7270150; doi:10.1038/s41598-020-65680-z)
Supplement: Supplementary file 1 — Supplementary information. [file 41598_2020_65680_MOESM1_ESM.docx]

**Supplementary Information**

**Differential miRNAs expression pattern of irradiated breast cancer cell lines is correlated with radiation sensitivity**

**Nastaran Masoudi-Khoram, Parviz Abdolmaleki*, Nazanin Hosseinkhan, Alireza Nikoofar, Seyed Javad Mowla, Hamideh Monfared, and Gustavo Baldassarre**

^1^ Department of Biophysics, Faculty of Biological Sciences, Tarbiat Modares University, Tehran, Iran

^2^ Endocrine Research Center, Institute of Endocrinology and Metabolism, Iran University of Medical Sciences (IUMS), Tehran, Iran

^3^ Department of Radiotherapy, Iran University of Medical Sciences (IUMS), Tehran, Iran

^4^ Department of Genetics, Faculty of Biological Sciences, Tarbiat Modares University, Tehran, Iran

^5^ Division of Experimental Oncology 2, Department of Translational Research, CRO, National Cancer Institute, Aviano, Italy

**Correspondence to:** **Parviz Abdolmaleki**

Department of Biophysics, Faculty of Biological Sciences, Tarbiat Modares University, P.O. Box 1415-154, Tehran, Iran.

**Tel:** +98 21-82883404

**E-mail**: parviz@modares.ac.ir

Supplementary Fig. S1.

Full length blot of Western blot analysis.

Full length Blot of Figure 3a.

| 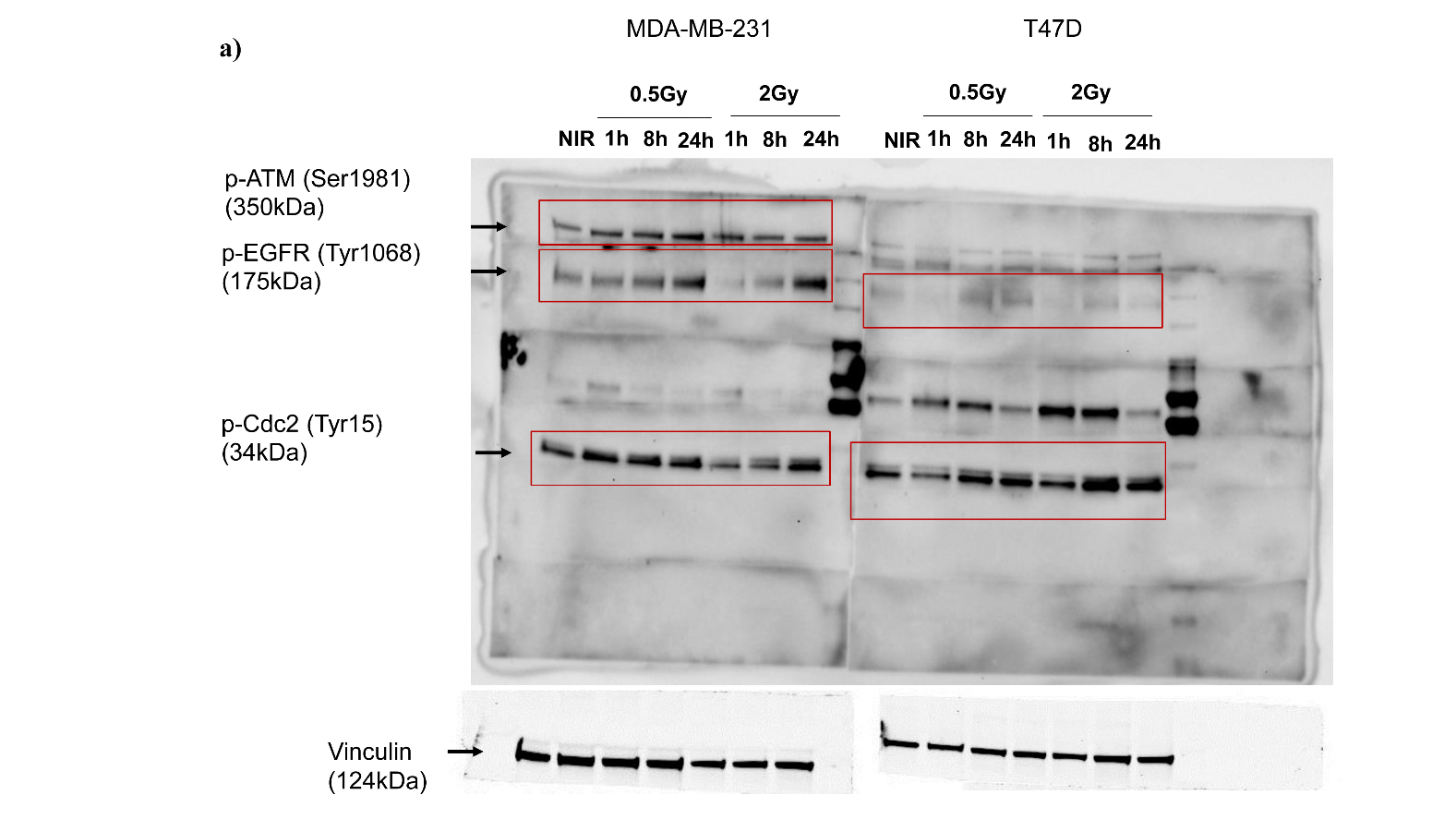 |
| --- |
| 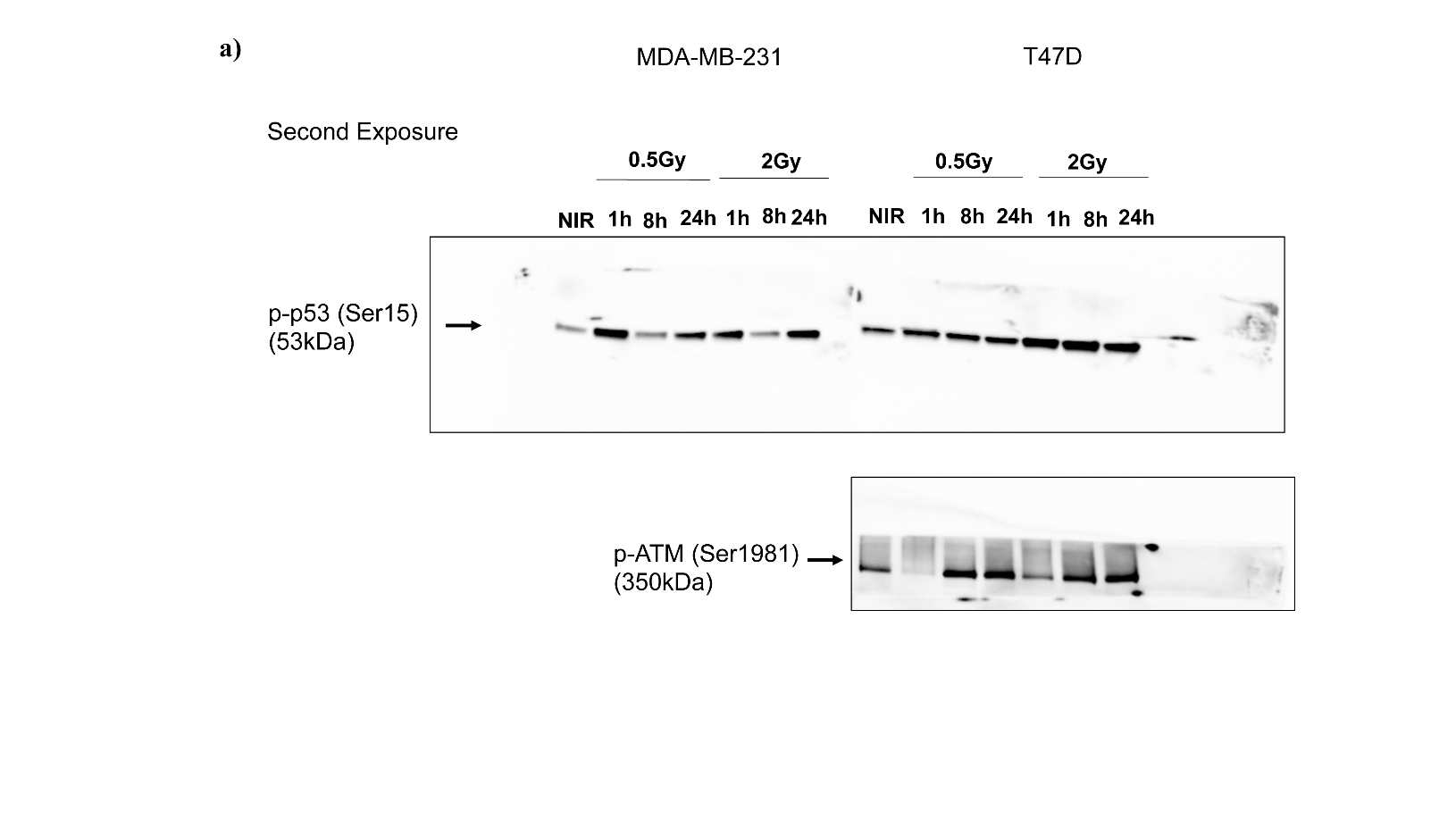 |

Full length Blot of Figure 3b.

| 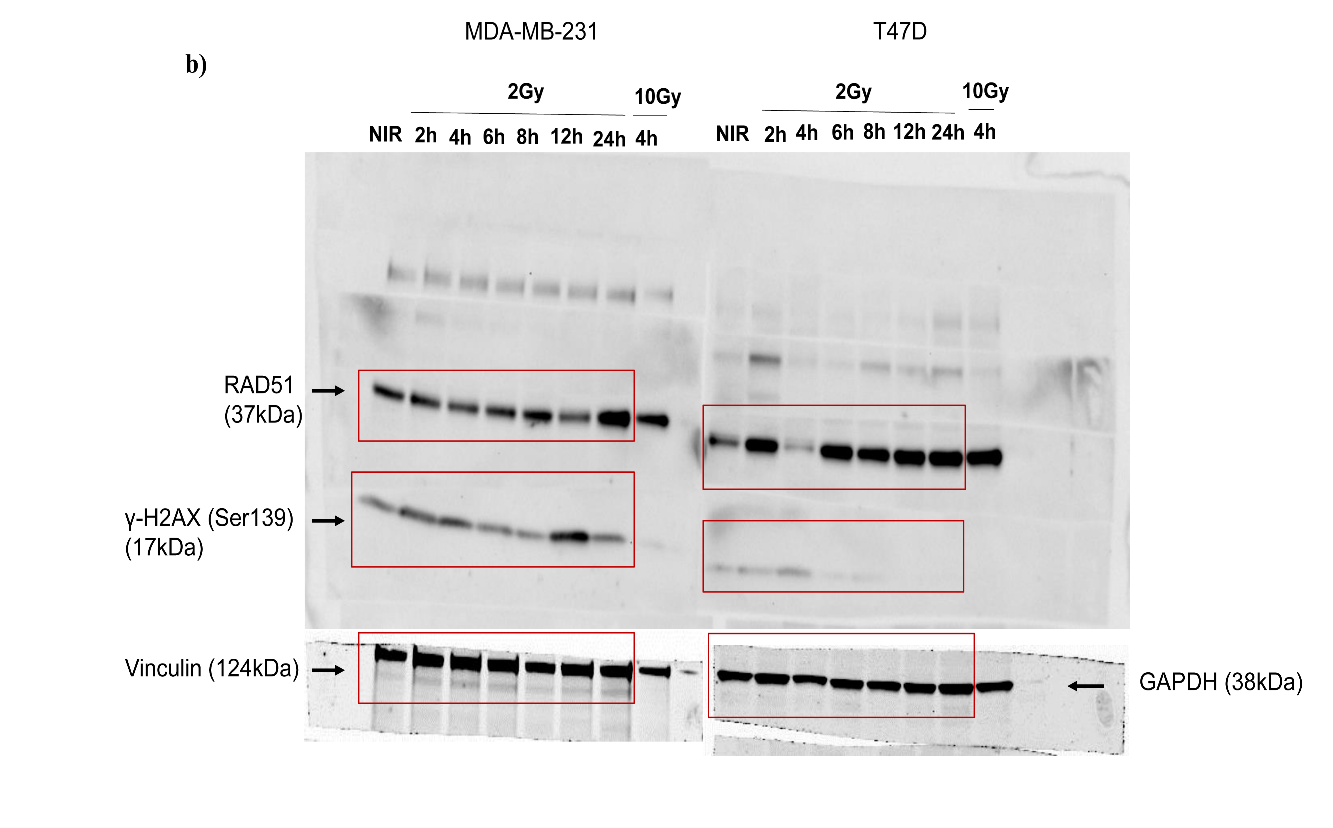 |
| --- |
